# UV-induced inhibition of adipokine production in subcutaneous fat aggravates dermal matrix degradation in human skin

**DOI:** 10.1038/srep25616

**Published:** 2016-05-10

**Authors:** Eun Ju Kim, Yeon Kyung Kim, Min-Kyoung Kim, Sungsoo Kim, Jin Yong Kim, Dong Hun Lee, Jin Ho Chung

**Affiliations:** 1 Department of Dermatology, Seoul National University College of Medicine, Seoul, Republic of Korea; 2 Laboratory of Cutaneous Aging Research, Biomedical Research Institute, Seoul National University Hospital, Seoul, Republic of Korea; 3 Institute of Human-Environment Interface Biology, Seoul National University, Seoul, Republic of Korea; 4 Institute on Aging, Seoul National University, Seoul, Republic of Korea

## Abstract

Ultraviolet (UV) exposure to the human skin reduces triglycerides contents and lipid synthesis in the subcutaneous (SC) fat. Because adiponectin and leptin are the most abundant adipokines from the SC fat, we aim to investigate how they interact with UV exposure and skin aging. The expressions of adiponectin and leptin were significantly decreased in SC fat of sun-exposed forearm skin, in comparison with that of sun-protected buttock skin of the same elderly individuals, indicating that chronic UV exposure decreases both adipokines. Acute UV irradiation also decreased the expressions of adiponectin and leptin in SC fat. The expressions of adiponectin receptor 1/2 and leptin receptor were significantly decreased in the dermis as well as in SC fat. Moreover, while exogenous adiponectin and leptin administration prevented UV- and TNF-α induced matrix metalloproteinase (MMP)-1 expression, they also increased UV- and TNF-α induced reduction of type 1 procollagen production. Silencing of adiponectin, leptin or their receptors led to an increased MMP-1 and a decreased type 1 procollagen expression, which was reversed by treatment with recombinant human adiponectin or leptin. In conclusion, UV exposure decreases the expression of adiponectin and leptin, leading to the exacerbation of photoaging by stimulating MMP-1 expression and inhibiting procollagen synthesis.

The subcutaneous (SC) fat of the skin is now appreciated as a dynamic organ which modulates diverse endocrine and metabolic functions through the release of a large number of bioactive substances, termed adipokines^1^,^2^. Adiponectin and leptin are the most abundantly expressed adipokines in the SC adipose tissues, and regulate multiple activities through endocrine, paracrine or autocrine mechanisms. In general, leptin levels are closely correlated with the fat mass/body mass index, whereas decreased adiponectin levels are associated with morbid states such as insulin resistance syndrome, supporting its role as an insulin sensitizing, anti-inflammatory, and anti-apoptotic mediator^3^. Regarding aging-related changes of fat and adipokines, levels of adiponectin and leptin are known to decrease in parallel with fat mass reduction with intrinsic aging^4^,^5^,^6^. However, their expression changes in SC fat from photoaged human skin, and potential roles in photoaging have not been determined.

Ultraviolet (UV) radiation from the sunlight is the most important contributor of photoaging^7^,^8^. Photoaging is characterized by morphological changes including deep wrinkles and loss of elasticity, as well as histological changes such as extracellular matrix alterations like damaged and disorganized collagen fibrils in the dermis. These alterations are the outcome of inhibition of procollagen synthesis and accelerated collagen breakdown by UV-induced matrix metalloproteinases (MMPs) secreted from epidermal keratinocytes and dermal fibroblasts^9^. Recently, we reported that although UV irradiation cannot penetrate into SC layer, acute and chronic UV exposure to the skin significantly reduces triglycerides contents and lipids synthesis in the human SC fat tissues^10^, suggesting subsequent alteration of adipokines upon UV exposure. Here, we investigated the influence of acute UV irradiation and photoaging on the productions of adiponectin and leptin in the SC fat, and whether these changes affect photoaging processes *vice versa*.

## Results and Discussion

### UV decreases adiponectin and leptin in SC fat tissues

**Figure 1:**
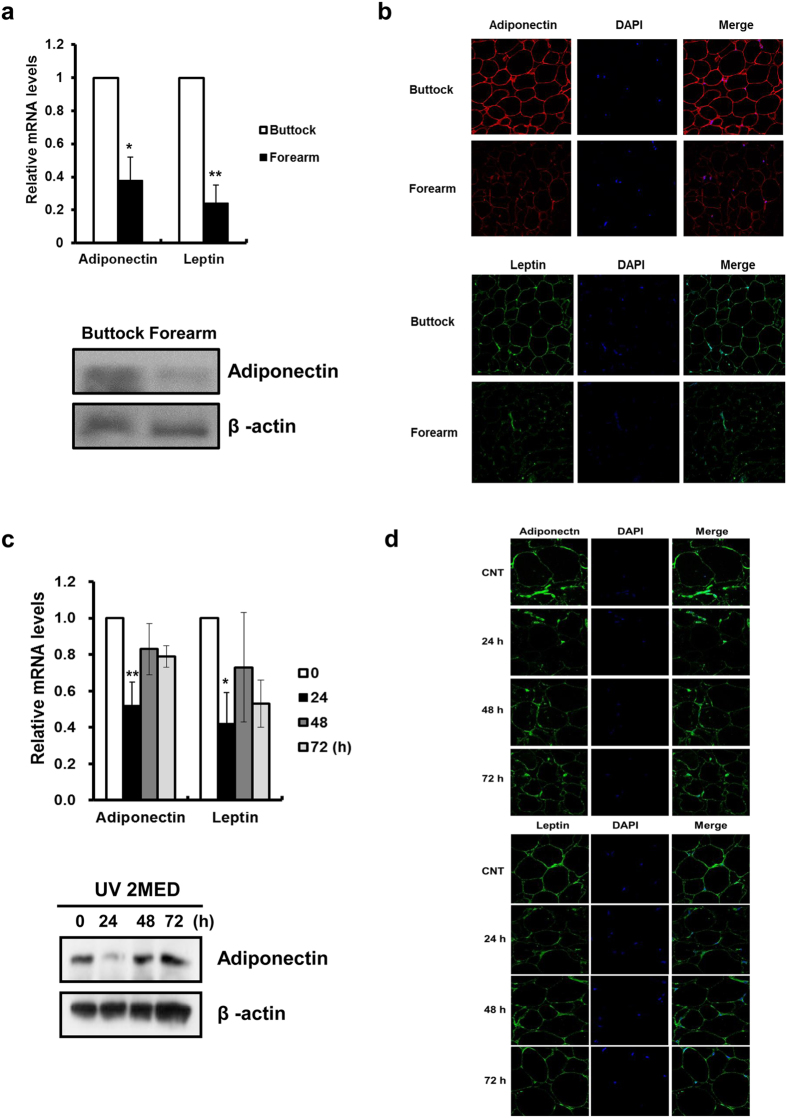
UV irradiation decreases the expression of adiponectin and leptin in the subcutaneous (SC) fat tissues of the human skin *in vivo.* Aged human (mean age 72.7 year; age range 70–75 years) buttock/forearm skin and young human (mean age 26.5 year; age range 21–33 years) buttock skin irradiated by 2 minimal erythema dose (MED) of ultraviolet (UV) light was obtained by punch biopsy, and SC fat tissues were separated from the dermis. (**a**,**b**) Adiponectin and leptin expression in the SC fat tissues in photoaged forearm and intrinsically aged buttock skin (n = 5). (**a**) mRNA and protein (Western blot), (**b**) immunofluorescent staining. (**c**,**d**) Adiponectin and leptin expression in the SC fat tissues following UV irradiation (n = 3). (**c**) mRNA and protein (Western blot), (**d**) immunofluorescent staining. Real-time PCR was used to determine mRNA of each gene (Data represent mean ± SEM of the ratio between each gene and 36B4. *P < 0.05, **P < 0.01). Cropped blots are used in this figure and the gels for Western blot have been run under the same experimental conditions.

Firstly, we compared the expression of adiponectin and leptin in the SC fat tissues of matched forearm (sun-exposed) and buttock (sun-protected) skin of healthy elderly Korean subjects. Aged human buttock and forearm skin was obtained by punch biopsy and SC fat tissues were separated from the dermis. The mRNA and protein expressions of adiponectin (*P* = 0.011) and leptin (*P* = 0.002) were significantly decreased in the SC fat tissues of forearm skin, in comparison with those of buttock skin (Fig. 1a,b). Basal leptin protein in SC fat could not be detected by Western blot analysis because of its very low expression in non-obese healthy volunteers. Our prior study revealed that decreased lipid levels in photodamaged forearm SC fat tissues was not attributed to anatomical site differences, but to the chronic UV irradiation, because SC fat tissues from the forearm skin of young volunteers did not show significant decrease in the expression of these adipokines (Supplementary Fig. 1), adipogenic transcription factors and lipogenic enzymes as well as lipid levels^10^, compared with those from the buttock skin of the same volunteers. Then we investigated expressions of these adipokines in acute UV-irradiated skin. Sun-protected buttock skin of young subjects was irradiated with 2 minimal erythema dose (MED) of UV. A single acute UV irradiation markedly decreased the expressions of adiponectin and leptin in SC fat tissues (Fig. 1c,d). The most remarkable reduction of adiponectin protein occurred at 24 hours post-irradiation (Fig. 1c). Previously, we demonstrated that acute exposure to UV dramatically decreased the mRNA expression of the transcription factors such as CCAAT/enhancer-binding protein (C/EBPα), peroxisome proliferator-activated receptor (PPAR)γ, and sterol regulatory element binding protein (SREBP)1c in the SC fat tissues^10^. The fat mass is regulated by alterations in both number (adipogenesis) and volume (lipogenesis) of adipocytes^11^, and adipogenesis is primarily controlled by key transcription factors such as C/EBPα, PPARγ, and SREBP-1c^12^,^13^,^14^. In addition, adiponectin is upregulated in response to C/EBPα, PPARγ and SREBP1 activation^15^,^16^,^17^. Therefore, our previous study and these results suggest that acute and chronic UV exposure decrease the production of adiponectin and leptin in the SC adipose tissues, at least in part, through downregulation of gene expression involved in adipogenesis.

### Exogenous adiponectin and leptin prevent UV- and TNF-α induced MMP-1 and TNF-α expression, as well as prevent UV- and TNF-α induced procollagen reduction

**Figure 2:**
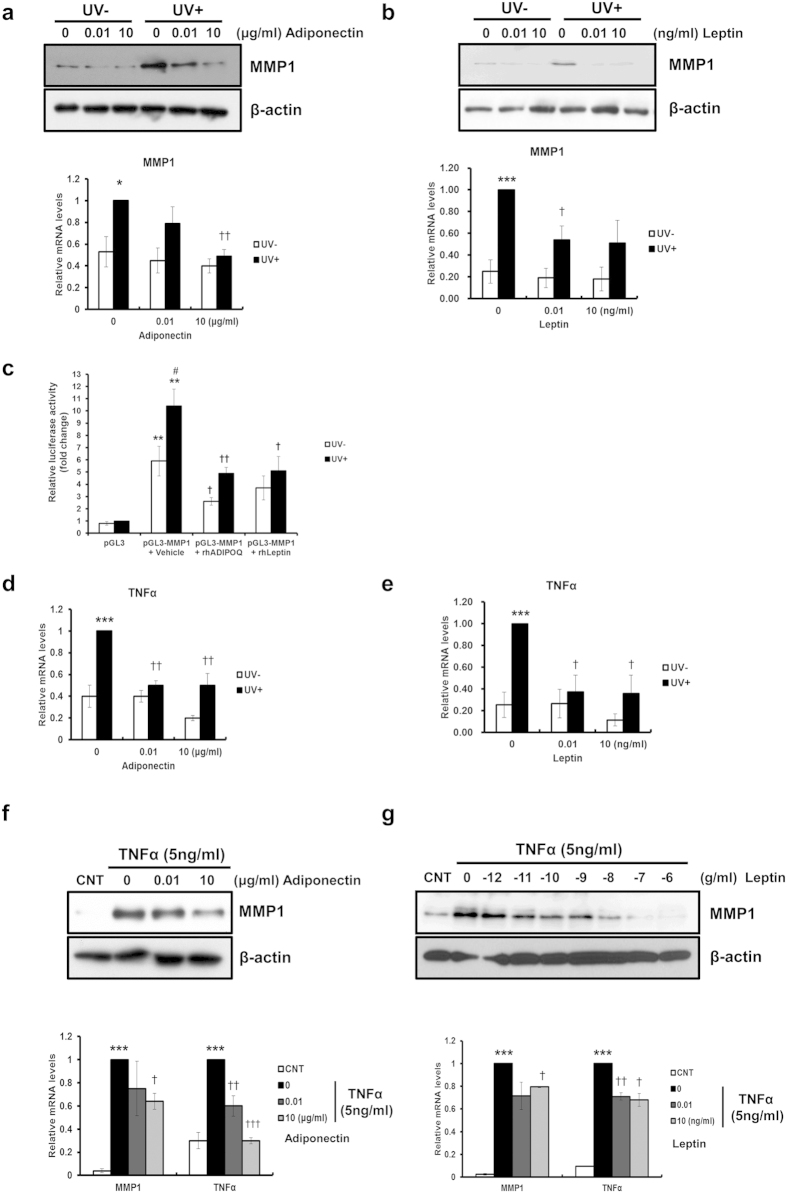
Exogenous adiponectin and leptin prevent UV- and TNF-α induced increases of MMP-1 expression in human dermal fibroblasts (HDFs). (**a**,**b**) Changes of UV-induced MMP-1 expressions following **(a)** adiponectin or **(b)** leptin administration (*P < 0.05, ***P < 0.001 vs. the UV − 0 group. ^†^*P* < 0.05, ^††^*P* < 0.01 vs. the UV + 0 group, n = 3). (**c**) Effect of UV and adipokines on the promoter activity of the MMP-1 gene (**P < 0.01 vs. the pGL3 group. ^†^*P* < 0.05, ^††^*P* < 0.01 vs. the pGL3-MMP1 + vehicle group, ^#^*P* < 0.05 vs. the UV- group, n = 5). (**d**,**e**) Changes of UV-induced TNF-α expressions following **(d)** adiponectin or **(e)** leptin administration (***P < 0.001 vs. the UV − 0 group. ^†^*P* < 0.05, ^††^*P* < 0.01 vs. the UV + 0 group, n = 3). (**f**,**g**) Exogenous adiponectin and leptin prevent TNF-α-induced MMP-1 expression in human dermal fibroblasts (HDFs). (**f**) Adiponectin and (**g**) leptin prevents TNF-α-induced MMP-1 mRNA (***P < 0.001 vs. the CNT group. ^†^*P* < 0.05, ^††^*P* < 0.01, ^†††^*P* < 0.001 vs. the TNFα + 0 group, n = 3) and protein expression as well as TNF-α mRNA. Data represent mean ± SEM of the ratio between each gene and 36B4. β-actin was detected from an equal volume of cell lysates as a loading control.

To elucidate the significance of reduced expressions of two major adipokines in skin photoaging, their effects on the expression of MMP-1, an important mediator of UV-induced skin damage, were examined. Human dermal fibroblasts (HDFs) were incubated with recombinant human adiponectin (0, 0.01 and 10 μg/ml) and leptin (0, 0.01 and 10 ng/ml) for 24 h after UV irradiation (100 mJ/cm^2^). Interestingly, while exogenous adiponectin and leptin slightly decreased the basal MMP-1 expression, they markedly prevented UV-induced MMP-1 in a dose-dependent manner in both mRNA and protein levels (Fig. 2a,b). Moreover, both adipokines transcriptionally inhibited UV-induced MMP-1 as demonstrated by the MMP-1 promoter assay (Fig. 2c). TNF-α is an important proinflammatory cytokine induced by UVB in fibroblasts as well as in keratinocytes, leading to the further progression of inflammatory cascade^18^ and matrix degradation^19^,^20^. Exogenous adiponectin and leptin administration also prevented UV-induced TNF-α in HDFs (Fig. 2d,e). Furthermore, when HDFs stimulated with TNF-α (5 ng/ml) were treated with adiponectin (0, 0.01 and 10 μg/ml) and leptin (10^−12^, 10^−11^, 10^−10^, 10^−9^, 10^−8^, 10^−7^, and 10^−6^ g/ml) for 24 h, adiponectin and leptin substantially prevented TNF-α-induced MMP-1 and TNF-α expression in HDFs in a dose-dependent manner (Fig. 2f,g).

**Figure 3:**
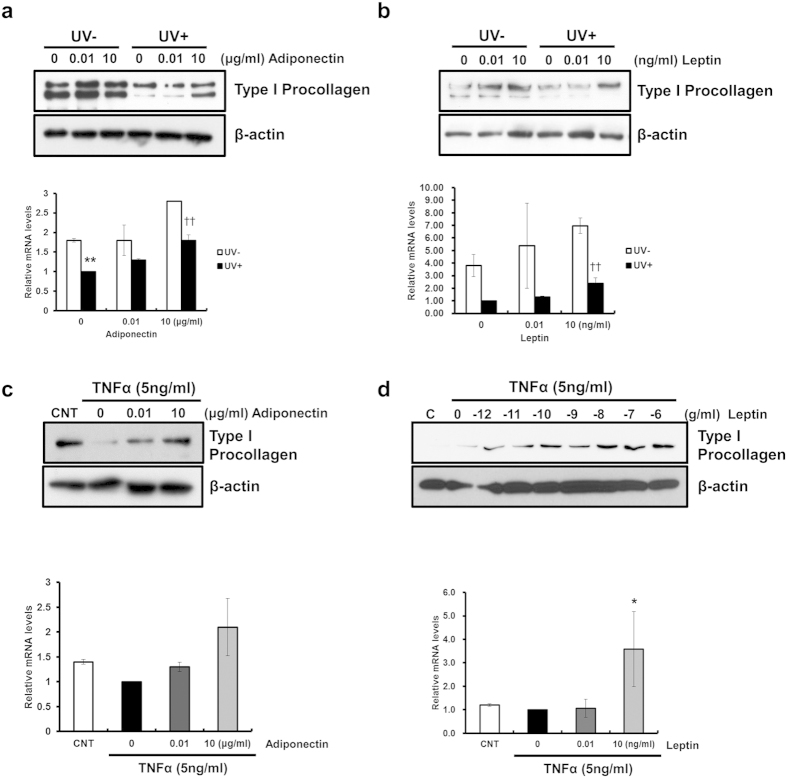
Exogenous adiponectin and leptin prevent UV- and TNF-α induced reductions of type I procollagen in human dermal fibroblasts (HDFs). **(a,b)** Changes of UV-induced type I procollagen expression following exogenous **(a)** adiponectin and **(b)** leptin administration for 24 h after UV irradiation. Levels of type I procollagen were determined by real-time PCR (n = 3, **P < 0.01, vs. the UV − 0 group. ^††^*P* < 0.01, vs. the UV + 0 group) and Western blot analysis. **(c,d)** Exogenous adiponectin (**c**) and leptin (**d**) prevents TNF-α-reduced type I procollagen expression in HDFs. HDFs were incubated with ADIPOQ (0, 0.01, and 10 μg/ml) and leptin (10^−12^, 10^−11^, 10^−10^, 10^−9^, 10^−8^, 10^−7^, and 10^−6^ g/ml) and TNF-α (5 ng/ml) for 24 h. Levels of type I procollagen were determined by real-time PCR (n = 3, *P < 0.05, vs. the CNT group) and Western blot analysis. Data represent mean ± SEM of the ratio between each gene and 36B4. β-actin was detected from an equal volume of cell lysates as a loading control.

On the other hand, UV-induced modulation of procollagen synthesis is also critical to loss of collagen and photoaging^21^. Thus we examined the effect of adiponectin and leptin on the procollagen expression. Exogenous adiponectin and leptin administration not only induced the expression of type I procollagen but also prevented UV- and TNF-α induced decreases of type I procollagen synthesis (Fig. 3a–d, respectively). Taken together, these data suggest that adiponectin and leptin may affect procollagen expression as well as MMP-1 expression.

### UV–induced downregulation of adiponectin and leptin receptors in dermis may be responsible for dermal matrix degradation

**Figure 4:**
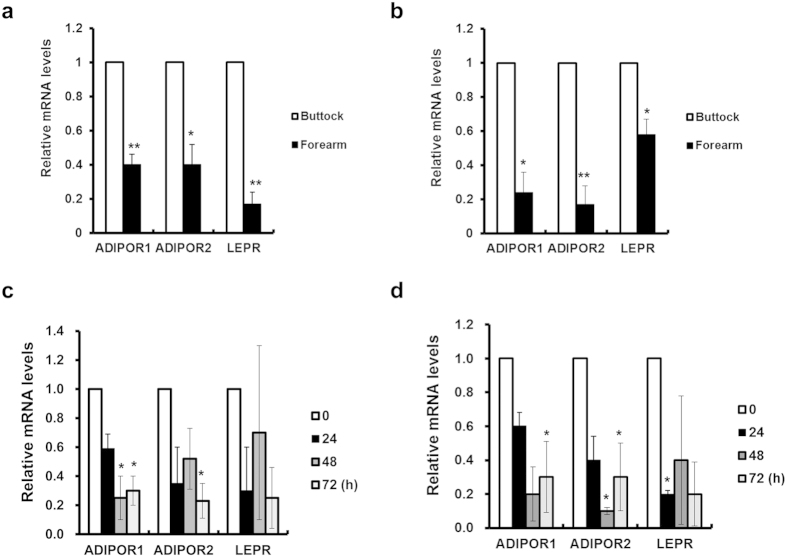
UV irradiation decreases the expression of adiponectin and leptin receptors in the dermis and the subcutaneous (SC) fat tissues of the human skin *in vivo.* Aged human (mean age 72.7 year; age range 70–75 years) buttock/forearm skin and young human (mean age 26.5 year; age range 21–33 years) buttock skin irradiated by 2 minimal erythema dose (MED) of ultraviolet (UV) light was obtained by punch biopsy, and SC fat tissues were separated from the dermis. (**a**–**d**) Adiponectin receptors (ADIPOR1 and ADIPOR2) and leptin receptor (LEPR) mRNA expression in the dermis (**a**,**c**) and in the SC fat tissues (**b**,**d**). Real-time PCR was used to determine mRNA of each gene (Data represent mean ± SEM of the ratio between each gene and 36B4. n = 3~5, *P < 0.05, **P < 0.01).

**Figure 5:**
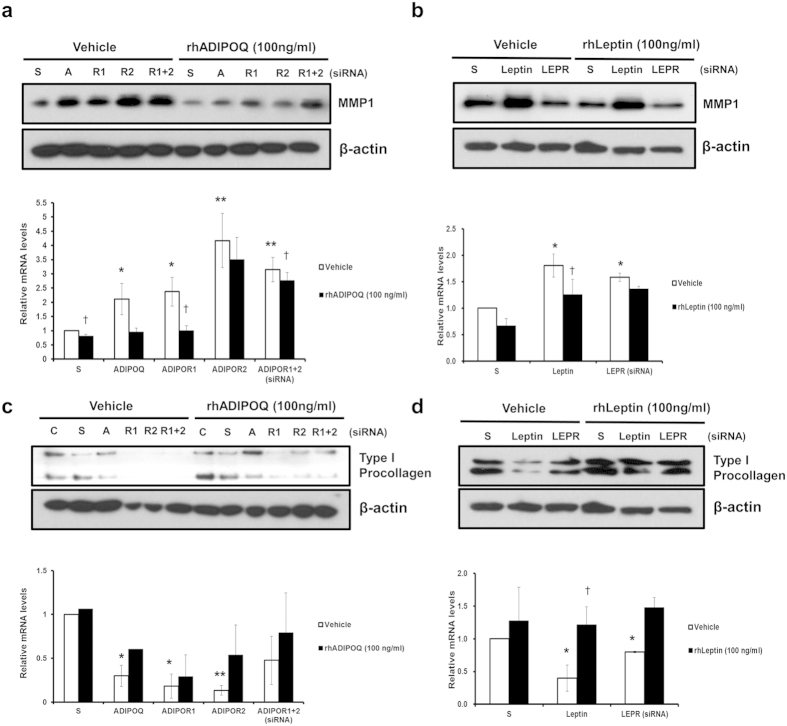
Adiponectin/leptin and its receptors silencing led to an increased MMP‐1, while a decreased procollagen expression, which was reversed by recombinant human adiponectin/leptin. (**a**,**b**) Changes of MMP-1 expression following silencing of (**a**) adiponectin, (**b**) leptin, or its receptors, and administration of recombinant human adiponectin (rhADIPOQ) or leptin. *P < 0.05, **P < 0.01 vs. S/vehicle group, ^†^*P* < 0.05, vs. each vehicle group, (n = 3). (**c,d**) Changes of type I procollagen expression following silencing of (**c**) adiponectin, (**d**) leptin, or its receptors, and administration of recombinant human adiponectin (rhADIPOQ) or leptin. *P < 0.05, **P < 0.01 vs. S/vehicle group, ^†^*P* < 0.05, vs. each vehicle group. (n = 3). C: control, S: scrambled siRNA, A, R1, R2: adiponectin, ADIPOR1, ADIPOR2 siRNA, LEPR: leptin receptor siRNA. Data represent mean ± SEM of the ratio between each gene and 36B4. β-actin was detected from an equal volume of cell lysates as a loading control. Cropped blots are used in this figure and the gels for Western blot have been run under the same experimental conditions.

Adiponectin binds to adiponectin receptor 1 (ADIPOR1) or adiponectin receptor 2 (ADIPOR2) to initiate its biological actions^22^, whereas leptin does to leptin receptor (LEPR). To investigate whether reduced expressions of adipokines in the skin aging process provoke dermal matrix degradation, we examined expression changes of their corresponding receptors in the skin. Both ADIPOR1 and ADIPOR2 were expressed in fibroblasts, dermis, and epidermis as well as adipocytes, but more abundant ADIPOR1 was expressed than ADIPOR2 (Supplementary Fig. 2). ADIPOR1, ADIPOR2 and LEPR were significantly decreased in the dermis (*P* = 0.009, *P* = 0.035, and *P* = 0.006, respectively) as well as SC fat tissues (*P* = 0.023, *P* = 0.002, and *P* = 0.016, respectively) of photoaged forearm skin, as compared with intrinsically aged buttock skin (Fig. 4a,b, respectively). Besides, acute UV irradiation markedly decreased expressions of ADIPOR1/2 and LEPR in the dermis and SC fat tissues *in vivo* (Fig. 4c,d, respectively). Our findings indicate that UV exposure may modulate functions of adiponectin and leptin via decreased expression of their corresponding receptors in the skin, particularly in the dermis, as well as inhibition of their *de novo* production in SC fat tissues. Moreover, the siRNA-mediated transient knockdown of adiponectin and its receptors (Supplementary Fig. 3a), especially ADIPOR2, increased the MMP-1 mRNA expression in HDFs (Fig. 5a). Besides, this increased expression of MMP-1 following knockdown of adiponectin and ADIPOR1, but not ADIPOR2, was reversed by treatment of recombinant human adiponectin (100 ng/ml), suggesting that the action of adiponectin in the HDFs may exert mainly through by adiponectin receptors (Fig. 5a). Similarly, leptin and its receptor silencing (Supplementary Fig. 3b) led to an increased level of MMP-1, which was reversed by recombinant human leptin (100 ng/ml) (Fig. 5b). Conversely, silencing of adiponectin, leptin or its receptors led to a decreased procollagen expression, which was effectively reversed by recombinant human adiponectin or leptin (Fig. 5c,d). Taken together, these data suggest that UV decreases the expression of adiponectin, leptin or its receptors, and these changes may aggravate MMP-1-mediated collagen destruction. In contrast with a traditional view as a passive lipid reservoir, adipose tissue is now considered as a dynamic endocrine organ that secretes adipokines, which play crucial physiological roles such as regulating body weight, energy homeostasis, immunity, hematopoiesis, angiogenesis, wound healing, osteogenesis, and even gastrointestinal functions^1^,^2^,^23^. However, little is known about the role of SC fat tissue and adipokines in photoaging, particularly in humans. Previous reports showed that UV exposure to mice reduced the levels of plasma adiponectin^24^,^25^ and those of adiponectin mRNA in ovarian fat^24^. Diverse agents have been reported to positively or negatively regulate adiponectin expression^26^,^27^. For instance, thiazolidinediones, synthetic ligands of PPARγ, increase both adiponectin gene expression in adipocytes and circulating adiponectin levels^16^. Insulin and insulin-like growth factor 1 also increase expression of adiponectin, whereas TNF-α decreases adiponectin gene expression, suggesting a relationship with TNF-α-induced insulin resistance^28^. Transcription of the leptin gene is activated during adipocyte differentiation^29^. UV-induced down-regulation of adipogenic transcription factors such as C/EBPα, PPARγ and SREBP1c^10^, which are known to promote adiponectin^15^,^30^ and leptin^31^,^32^,^33^,^34^ expression, may be responsible for reduced adiponectin and leptin synthesis by UV in SC fat tissues. Moreover, several cytokines such as IL-6 and TNF-α are released after UV irradiation^35^,^36^ and can reduce the expression of adipokine receptors^37^. In addition to demonstrating that adiponectin and leptin were down-regulated in photoaged and acutely UV irradiated skin, we found that adiponectin and leptin have unexpected function implicated in skin biology which may protect dermal matrix degradation during skin aging by preventing MMP-1 increases and collagen decline.

Controversy still exists in the effects of adiponectin and leptin on the expression of MMPs and collagen. While Ezure and Amano reported that adiponectin increased collagen and hyaluronic acid production in dermal fibroblasts^38^, others showed that adiponectin inhibited collagen gene expression and myofibroblast differentiation in normal and scleroderma fibroblasts^39^. More recently, Nakasone *et al.* showed that adiponectin induced MMP-1 and MMP-3 mRNA, but did not significantly affect type 1 collagen level mRNA in dermal fibroblasts, suggesting high levels of adiponectin could modulate dermal fibrosis in recipients with chronic graft-versus-host diseases^40^. One possible explanation for the discrepancy between the present study and others is differences in experimental conditions such as serum deprivation and the nature of the samples.

Consistent with our findings, leptin inhibited the expression of MMP-1 in LX-2 hepatic stellate cells^41^ and that of MMP-2 in cardiac myofibroblasts^42^, and increased the expression of procollagen I in neonatal rat cardiac myofibroblasts^43^, cardiac myofibroblasts^42^, and vascular smooth muscle cells^44^. Various diseases such as stroke, coronary heart disease, steato-hepatitis, insulin resistance, nonalcoholic fatty liver disease, and some cancers are associated with reduced adiponectin level. Systemic implications and possible mechanisms of adipokine changes following UV irradiation and photoaging warrant further investigations. Besides, further studies are required to elucidate the interactions between adipokines and elastin, which is another crucial player in photoaged skin.

In conclusion, reduced adiponectin and leptin in the SC fat, and their receptors in UV-irradiated and photoaged skin may lead to the exacerbation of photoaging by stimulating MMP‐1 expression and inhibiting procollagen synthesis.

## Methods

### Human studies

The elderly (mean age 72.7 years, age range 70–75 years) and young (mean age 35.3 year; age range 34-38 years) Koreans without current or prior skin disease provided both sun-protected buttock and photo-damaged extensor forearm skin samples. Another group of young volunteers (mean age 26.5 years, age range 21–33 years) provided buttock skin samples^10^. The buttock skin was irradiated with a F75/85 W/UV21 fluorescent lamp with an emission spectrum between 275 and 380 nm (peak at 310–315 nm). The buttock skin was irradiated with UV light filtered through a Kodacel filter (TA401/407; Kodak) to remove wavelengths below 290 nm (UVC). The minimal erythema dose (MED) was determined 24 h after irradiation. The MED usually ranged between 70 and 90 mJ/cm^2^ for the brown skin of Koreans. We used 2 MED in this study. Irradiated and non-irradiated buttock skin samples were obtained from each subject by punch biopsy, and the SC fat tissues were manually separated from the dermis with liberal margins. This study was approved by the Institutional Review Board at Seoul National University Hospital (IRB No. 1504-110-667), and all subjects provided written informed consent. The study was conducted in accordance with the Principles of the Declaration of Helsinki.

### Western blot analysis and immunofluorescence staining

The SC fat tissues were obtained and homogenized, and proteins were extracted using RIFA buffer (Upstate Biotechnology, Inc., Lake Placid, NY) containing complete protease, phosphatase inhibitor (Roche, Indianapolis, IN), 5 mM PMSF, and 1 mM DTT. The protein content was determined using the Bradford reagent (Bio-Rad, Hercules, CA). Equal amounts (50 μg) of protein were loaded, transferred and analyzed by Western blot analysis using a mouse polyclonal antibody against adiponectin. As a control, the level of β-actin was determined in each cell lysate using a goat polyclonal antibody for β-actin (Santa Cruz Biotechnology, Santa Cruz, CA).

For immunofluorescence staining, skin specimen sections (4 μm) were stained with primary mouse polyclonal antibody against adiponectin, and a goat polyclonal antibody against leptin (R&D Systems, Inc., Minneapolis, MN) in a humidified chamber at 4 °C for 18 h. After washing in PBS, the sections were incubated with a secondary Alexa 594 or Alexa 488-conjugated goat or mouse anti-rabbit IgG (Invitrogen, Life Technologies, Inc., Carlsbad, CA) antibody for 1 h at room temperature. The nuclei were counterstained with DAPI staining.

### Real-time and semi-quantitative polymerase chain reaction (RT-PCR)

Total RNA was prepared from separated subcutaneous fat tissues using the Trizol method (Life Technologies, Inc.) and 1 μg of total RNA was converted to cDNA using the First Strand cDNA Synthesis Kit (MBI Fermentas, Hanover, MD) according to the manufacturer’s instructions. To quantitatively estimate the mRNA expression of each gene, PCR was performed on a 7500 Real-time PCR System (Applied Biosystems, Life Technologies, Inc.) using SYBR^®^ Premix Ex Taq^TM^ (Takara Bio Inc., Shiga, Japan) according to the manufacturer’s instructions. Primer information is shown in Supplementary Table 1. The PCR conditions were 50 °C for 2 minutes, 95 °C for 2 minutes, followed by 40 cycles at 95 °C for 15 s and 60 °C for 1 minute. The data are presented as fold changes in gene expression normalized to 36B4.

### Cell studies

Primary human dermal fibroblasts (HDFs), isolated from foreskin, were cultured in DMEM with 10% FBS (Gibco, Thermo Fisher Scientific, Inc., Waltham, MA) and antibiotics. Cultured HDFs at passages 6–10 were used for the experiments as previously described^10^. To determine the effect of adiponectin and leptin on MMP-1 expression, the HDFs were incubated with recombinant human adiponectin (0.01 and 10 μg/ml, BioVision, Inc., Milpitas, CA) and leptin (0.1 and 100 ng/ml, BioVendor, Asheville, NC) only, or with recombinant human adiponectin (0.01 and 10 μg/ml) and leptin (0.1 and 100 ng/ml) after UV irradiation (100 mJ/cm^2^), and with TNF-α (5 ng/ml), and recombinant human adiponectin (0.01 and 10 μg/ml) and leptin (10^−12^, 10^−11^, 10^−10^, 10^−9^, 10^−8^, 10^−7^, and 10^−6^ g/ml) for 24 h and then supernatant and cells were harvested. Activation of endogenous MMP-1 does not practically occurred in human dermal fibroblast cultures^45^. The amount of proMMP-1 and type I procollagen proteins secreted into culture media were analyzed using rabbit polyclonal antibodies against MMP-1 (Lab Frontier, Seoul, Korea) and monoclonal anti-pro αl(1)-N-propeptide antibody (SP1.D8; Developmental Studies Hybridoma Bank, Iowa City, IA, USA), respectively, as previously described^46^,^47^.

### Plasmid constructs, transient transfection, and luciferase reporter assay

The human MMP-1 promoter/luciferase plasmids (MMP-1959luc) contained the firefly luciferase gene under the transcriptional control of the human MMP-1 promoter in the pGL3 basic reporter vector (Promega, Madison, WI, USA). For luciferase assays, HDFs were cultured in 6-well plates for 2 days before transfection. MMP1-Luc were transiently co-transfected into cells using Lipofectamine 2000 (Invitrogen). At 24-h post-transfection, cells were UV-irradiated with/without 100 ng/ml of recombinant human adiponectin or leptin. After 24 h, cells were lysed and analyzed for luciferase activity. The pRL-TK plasmid was used as an internal control for transfection efficiency^48^.

### RNA interference

Endogenous adiponectin, adiponectin receptor 1 and 2 (ADIPOR1/2), and leptin and leptin receptor in the HDFs were depleted through two sequential transfections. A total of 100 nM adiponectin, ADIPOR1/2, leptin and leptin receptor siRNA, 100 nM scrambled control siRNA or 100 nM GAPDH siRNA were transfected into the HDFs using Lipofectamine 2000. To exclude possible off-target effect of siRNA, different sequences of adiponectin- and leptin-targeting siRNAs (n = 3 and n = 2, respectively) were employed. After 24 h of transfection, the cells were treated with 100 ng/ml of recombinant human adiponectin or leptin, and harvested after 24 h.

### Statistical analysis

Data are presented as the means ± standard error of the mean (SEM). Significance was analyzed by the Paired *t*-test or Student’s *t*-test. When several groups or timepoints were compared, two-way ANOVA or one-way ANOVA followed by Bonferroni post-hoc tests were employed. Differences were considered significant when P < 0.05.

## Additional Information

**How to cite this article**: Kim, E. J. *et al.* UV-induced inhibition of adipokine production in subcutaneous fat aggravates dermal matrix degradation in human skin. *Sci. Rep.* **6**, 25616; doi: 10.1038/srep25616 (2016).

## Supplementary Material

Supplementary Information
