# Supplementary material for: UV-induced inhibition of adipokine production in subcutaneous fat aggravates dermal matrix degradation in human skin
Source: Sci Rep. 2016 May 10;6:25616. doi: 10.1038/srep25616 (PMC4861907; doi:10.1038/srep25616)
Supplement: Supplementary Information [file srep25616-s1.pdf]

Supplementary Information

UV-induced inhibition of adipokine production in subcutaneous fat aggravates dermal matrix degradation in human skin

Eun Ju Kim[1,2,3], Yeon Kyung Kim[1,2,3], Min-Kyoung Kim[1,2,3], Sungsoo Kim[1,2,3], Jin Yong Kim[1,2,3], Dong Hun Lee[1,2,3*], and Jin Ho Chung[1,2,3,4*]

Supplementary Table 1. Primer information

Gene	Forward	Reverse	Size (bp)	GenBank (gene ID)
hAdiponectin	TTC AGG TGC ATT CCA CCA	TCC TCC ATG GGT TTT GCA	530	9370
hAdiponectin Receptor 1	CCT GCC AGT AAC AGG GAA	AGG AGA AGC TGA GGC AGA	499	51094
hAdiponectin Receptor 2	ATC CCA TGA ACG AGC CAA	GGT GTC CAT GCA AGA GGA	363	79602
hLeptin	GCT GAA CAG CCA AAT GCA	TGC ATC TCC ACA CAC CAA	398	3952
hLeptin receptor	CCT ATG AGG ACG AAA GCC AGA	GAA TAA ACA GGG GGC TGG GAA	392	3953
hMMP-1	AAG CGT GTG ACA GTA AGC TA	AAC CGG ACT TCA TCT CTG	199	4312
hProcollagen α1	CTC GAG GTG GAC ACC ACC CT	CAG CTG GAT GGC CAC ATC GG	366	1277
hTNFα	TCC TTC AGA CAC CCT CAA CC	AGG CCC CAG TTT GAA TTC TT	173	7124
h36B4	TCG ACA ATG GCA GCA TCT AC	TGA TGC AAC AGT TGG GTA GC	130	6175

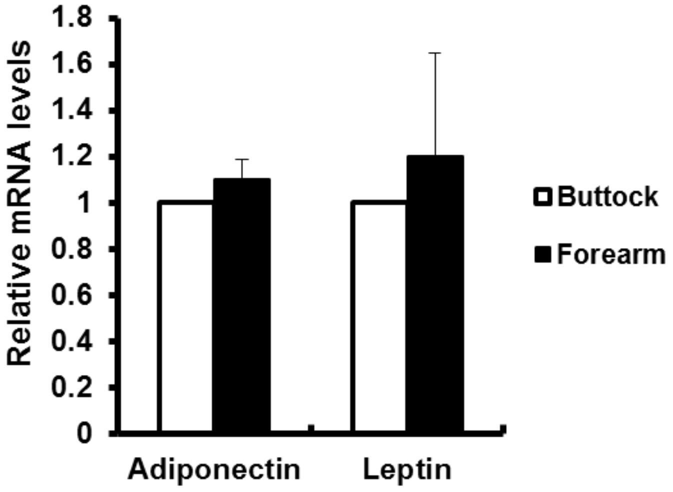

Supplementary Figure 1. **The expression of adiponectin and leptin in the subcutaneous (SC) fat tissues of the young human skin *in vivo.*** Young human (mean age 35.3 year; age range 34-38 years) buttock/forearm skin was obtained by punch biopsy, and SC fat tissues were separated from the dermis. Real-time PCR was used to determine mRNA of each gene (Data represent mean±SEM of the ratio between each gene and 36B4. n=3).

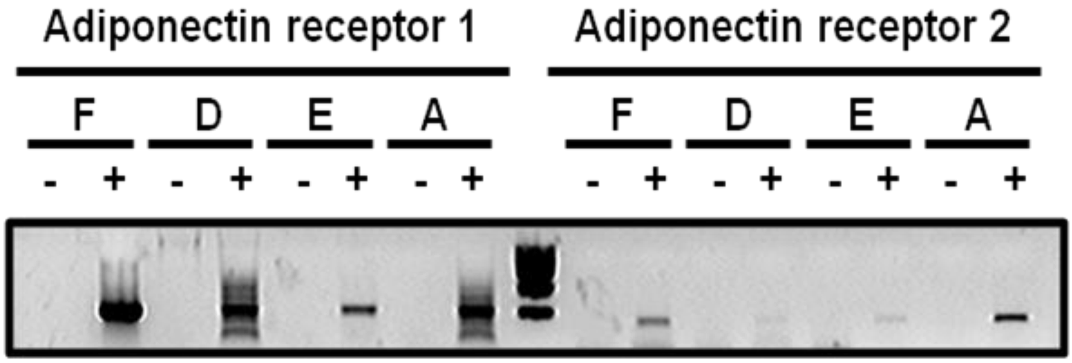

Supplementary Figure 2. Validation of adiponectin receptors expression in human skin. Adiponectin receptors mRNA levels were measured by semi-quantitative RT-PCR. F : fibroblasts, D : dermis, E : epidermis, A : adipocytes. - : without cDNA, + : cDNA from 1µg of total RNA.

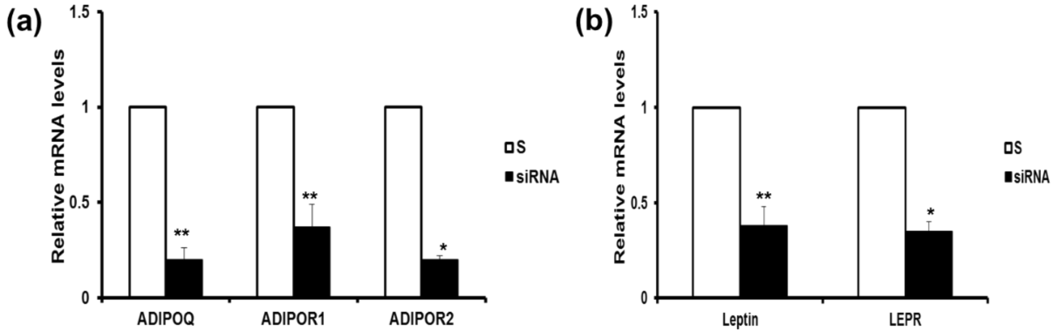

Supplementary Figure 3. Adiponectin or leptin and its receptors silencing system. HDFs were transfected with scrambled control siRNA and adiponectin, ADIPOR1, and ADIPOR2 (a), or leptin and LEPR (b) siRNA at 100 nM using Lipofectamine. Real-time PCR was used to determine mRNA of each gene (Data represent mean±SEM of the ratio between each gene and 36B4. n=3~5, *P<0.05, **P<0.01). S:scrambled siRNA, LEPR : leptin receptor siRNA.
